# Construction of a novel Wheat 55 K SNP array-derived genetic map and its utilization in QTL mapping for grain yield and quality related traits

**DOI:** 10.3389/fgene.2022.978880

**Published:** 2022-08-26

**Authors:** Xiaoli Fan, Xiaofeng Liu, Bo Feng, Qiang Zhou, Guangbing Deng, Hai Long, Jun Cao, Shaodan Guo, Guangsi Ji, Zhibin Xu, Tao Wang

**Affiliations:** ^1^ Chengdu Institute of Biology, Chinese Academy of Sciences, Chengdu, China; ^2^ University of Chinese Academy of Sciences, Beijing, China; ^3^ Yibin University, Yibin, China; ^4^ Innovative Academy for Seed Design, Chinese Academy of Sciences, Beijing, China

**Keywords:** grain yield, moisture content, wheat, genetic map, nutritional content

## Abstract

Wheat is one of the most important staple crops for supplying nutrition and energy to people world. A new genetic map based on the Wheat 55 K SNP array was constructed using recombinant inbred lines derived from a cross between Zhongkemai138 and Kechengmai2 to explore the genetic foundation for wheat grain features. This new map covered 2,155.72 cM across the 21 wheat chromosomes with 11,455 markers. And 2,846 specific markers for this genetic map and 148 coincident markers among different maps were documented, which was helpful for improving and updating wheat genetic and genomic information. Using this map, a total of 68 additive QTLs and 82 pairs of epistatic QTLs were detected for grain features including yield, nutrient composition, and quality-related traits by QTLNetwork 2.1 and IciMapping 4.1 software. Fourteen additive QTLs and one pair of epistatic QTLs could be detected by both software programs and thus regarded as stable QTLs here, all of which explained higher phenotypic variance and thus could be utilized for wheat grain improvement. Additionally, thirteen additive QTLs were clustered into three genomic intervals (C4D.2, C5D, and C6D2), each of which had at least two stable QTLs. Among them, C4D.2 and C5D have been attributed to the famous dwarfing gene *Rht2* and the hardness locus *Pina*, respectively, while endowed with main effects on eight grain yield/quality related traits and epistatically interacted with each other to control moisture content, indicating that the correlation of involved traits was supported by the pleotropic of individual genes but also regulated by the gene interaction networks. Additionally, the stable additive effect of C6D2 (*QMc.cib-6D2* and *QTw.cib-6D2*) on moisture content was also highlighted, potentially affected by a novel locus, and validated by its flanking Kompetitive Allele-Specific PCR marker, and *TraesCS6D02G109500*, encoding aleurone layer morphogenesis protein, was deduced to be one of the candidate genes for this locus. This result observed at the QTL level the possible contribution of grain water content to the balances among yield, nutrients, and quality properties and reported a possible new locus controlling grain moisture content as well as its linked molecular marker for further grain feature improvement.

## 1 Introduction

Wheat (*Triticum aestivum* L.) as one of the most important staple foods, accounts for 20% of global calorie and protein intake (FAO, http://www.fao.org/faostat/). Starch and protein, as the two major compositions of wheat grain, account for 60%–75% and 8%–20% of the total dry mass of the mature grain (Šramková et al., 2009), respectively, greatly influencing flour processing and end-use attributes, such as gelatinization temperatures and tensile properties (Branlard et al., 2001; Zhu et al., 2009; Shevkani et al., 2017), as well as affecting nutrient concentrations, ultimately determining food nutrition supply amount. The famous starch and protein biosynthetic genes including *SUT* (encoding sucrose transporter), *Wx* (encoding granule-bound starch synthase), *SsI* (encoding soluble starch synthases), *GPC* (encoding NAC transcription factor *Grain Protein Content*) (Murai et al., 1999; Aoki et al., 2002; Hurkman et al., 2003; Uauy et al., 2006; Avni et al., 2014) have been well studied for wheat quality improvement.

Moisture content typically determines grain storability. Grain with a low moisture content (<12%) is more resistant to storage, but too low may result in poor seed viability (Al-Yahya, 2001; Karunakaran et al., 2001). Additionally, as one of the three components with the highest proportion in the seed (Al-Mahasneh and Rababah, 2007), moisture content has a balance with protein and starch content, also influencing nutritive value and several processing quality parameters (Fang and Campbell, 2003), especially grain hardness and test weight parameters, which are widely employed as key indicators in wheat grading and pricing (Schuler et al., 1995; Cabral et al., 2018), because both of them can predict the grain milling efficiency and flour production (Muhamad and Campbell, 2004) and are also involved with the level of starch and protein content (Anjum and Walker, 1991). The related controlling genes for hardness (*Pina*, *Pinb*) have been well studied (Bhave and Morris, 2008; Qamar et al., 2014). However, the genetic factors controlling moisture content or test weight are less well discovered.

These grain traits generally present a correlation with grain yield and thus are generally noticed in the genetic improvement process, especially the well-known trade-offs between protein content and grain yield (Michel et al., 2019; Sandhu et al., 2021). However, with no change in concentration, the absolute production of these grain inner compositions should positively correlate with the total grain yield, and thus the absolute output of these compositions also deserves attention and research. In particular, with the advancement of processing and purification technology, it is now more convenient to extract wheat grain protein, starch or other nutrient substances (Day et al., 2006), both of which can be used as nutrition for functional food production or as raw materials for producing gluten, resistant starch, monosodium glutamate, fermentation substrate, etc. (Mohamed and Rayas-Duarte, 2003; Day et al., 2006; Xie et al., 2008; Ortolan and Steel, 2017; Sardari et al., 2019). They have been widely used in the food and processing industries, and thus not only the proportions of inner grain compositions but also their absolute yield are also factors in determining wheat’s commercial potential.

All these traits mentioned above are quantitative traits. Besides the known genes, previous studies have reported numerous QTLs providing genetic basis for these traits (Groos et al., 2003; Narasimhamoorthy et al., 2006; Zhang et al., 2011; Bonneau et al., 2013; Heo and Sherman, 2013; Kumar et al., 2019; Guo et al., 2020; Tu and Li, 2020; Colasuonno et al., 2021; Fradgley et al., 2022; Gudi et al., 2022). Although epistatic QTLs, which are involved in regulatory networks, are also crucial foundations for controlling complex traits, the majority of reported loci are additive QTLs (Xing et al., 2002; Malmberg and Mauricio, 2005). To date, several software programs have been developed to conduct both additive and epistatic QTL analyses, such as QTLNetwork (Yang et al., 2008) and IciMapping (Meng et al., 2015), both of which are helpful for determining the loci with main and interaction effects on the target traits.

With the development of the wheat genome, several arrays with high density SNP markers have been developed for wheat genetic research, such as 660, 90, and 55 K, etc. (Wang et al., 2014; Cui et al., 2017; Liu et al., 2018; Sun et al., 2020). These SNP arrays and their transformed KASP/CAPS markers provide great help for wheat genetic improvement. In this study, we constructed a new genetic map using a Wheat 55K array to complement wheat genomic information, and used this map to perform QTL analysis for grain features such as starch content, protein content, test weight and hardness of wheat grain, and to resolve their relationships with grain yield and grain nutrition yield at the QTL level.

## 2 Materials and methods

### 2.1 Plant materials and field trials

An F_6:7_ recombinant inbred line (RIL) population with 152 lines derived from a cross between varieties Zhongkemai138 (ZKM138) and Kechengmai2 (indicated as ZK-RILs) was used in this study. ZKM138 and KCM2 are both widely adaptable varieties in southwestern China and released by Chengdu Biology of Institute, CAS, (CIBCAS) in recent years. ZK-RILs and their parents were evaluated at Chengdu (30◦57′ N, 104◦94′ E) and Deyang (31◦13′ N, 104◦16′ E) in 2016–2017 and 2017–2018. A total of four environments were designated, namely, 17CD, 17DY, 18CD and 18DY. For each environment, the materials were planted in two replicated blocks. Each block contained two rows that were 2 m long and 0.25 m apart, and 20 seeds were evenly planted in each row. All of the recommended agronomic practices were followed in each trial.

### 2.2 Phenotypic evaluation and statistical analysis

Five representative plants in the centre of the rows were randomly sampled at physiological maturity for phenotypic evaluation. The lines were naturally air dried at sunny days and then dried at 42°C for 5–7 days, until samples presented constant weight. Three grain nutrition concentration related traits, including protein content (PC), starch content (SC), and moisture content (MC), and three grain grading related traits, including test weight (TW), grain hardness (GH) and water absorption (ABS), were measured by near-infrared reflectance spectroscopy (NIRS) with a Perten DA-7250 instrument (Perten Instruments, Huddinge, Sweden) and expressed on a 14% moisture basis. The grain yield per plant (GY) was weighed and used to calculate three physical compositions of the yield, including grain protein weight per plant (GPW = GY×PC×100), grain starch weight per plant (GSW = GY×SC×100) and grain water weight per plant (GWW = GY×MC×100). The spike length and kernel length were evaluated at maturity in the 18CD environment. The spike length was measured from the base of the rachis to the tip of the terminal spikelet excluding awns. The kernel length was investigated by lining up 20 kernels length-wise along a ruler with a precision of 0.1 mm.

### 2.3 Map construction

Genomic DNA for the 152 RILs and their parental lines was extracted from tender leaves using the TiangenTM Plant Genomic DNA Kit, and quality was evaluated using an agarose gel. The concentration was detected using the a Thermo Scientific NanoDrop 2000. The qualified DNA was genotyped using the Affymetrix 55K SNP array by Compass Biotechnology Company (Beijing, China) for genetic map construction and QTL mapping. Chip genotyping was performed according to the Axiom R 2.0 Assay for 384 Samples User Manual (Cui et al., 2017). The reported primers and their corresponding PCR system were used to detect *Rht2* (Ellis et al., 2002; Zhang et al., 2006) and *Pina* (Li et al., 2006).

Biallelic pleomorphic SNPs with >10% missing data and *p* < 0.01 by chi-square test of segregation distortion (departure from the expected 1:1 segregation ratio) were removed, and the remaining high-quality SNPs were binned by their pattern of segregation using the BIN function of IciMapping 4.1. Each bin had several markers; the correlation coefficient between them was 1, and one marker with the lowest missing rate was chosen to represent this bin. If there were no missing data of the markers in one bin, one marker was chosen randomly. Markers were tested for significant segregation distortion using a chi-square test. SNPs were sorted into groups using the MAP function in IciMapping 4.1. A logarithm of the odds (LOD) score of 3.5 and a recombination fraction of 0.4 were used to sort the SNPs with the Kosambi mapping function. Groups were ordered with the Kosambi mapping function within JoinMap v. 4.0, using an LOD score ≥3 after preliminary analysis of SNPs with LOD scores ranging from 2 to 10. The polarity of each chromosome was identified from the IWGSC wheat survey sequence, and groups were oriented to have the short arm above the long arm.

The SNP flanking sequences mapped in the ZK-RILs map were used to perform BLAST searches against (E value cutoff of 10–5) the IWGSC wheat survey contig sequences and the wheat genome assembly of *T. aestivum* cv. Chinese spring (CS) (IWGSC RefSeq v1.1, ftp://ftp.ensemblgenomes.org/pub/plants/release-44/fasta/triticum_aestivum/) to get their physical locations. The flanking sequences of SNPs and their best matched contigs were further used to blast against CDS of IWGSC RefSeq v1.1 to identify the number of coding-region SNPs (cSNPs) and perigenic SNPs (pSNPs) and intergenic SNPs (iSNPs), respectively (Cui et al., 2017; Liu et al., 2018). In addition, contig sequences to which the SNPs were best hits were screened in a BLASTN search against the coding sequences (CDSs) of Brachypodiun, barley, rice and maize. All CDSs were downloaded from http://plants.ensembl.org/index.html. An expectation value (E) of 1E-10 was defined as the significance threshold. Synteny analyses with common wheat, Brachypodiun, barley, rice and maize genomes were performed based on the SNP orders in the ZK genetic map and on the corresponding CDSs in the genome sequences of Brachypodiun, barley, rice and maize genomes. The SNPs flanking sequences of 90 K (Wang et al., 2014) and 660 K (Cui et al., 2017) were used to blast against IWGSC RefSeq v1.1 assembly sequences to map their physical locations, and compared with the SNP locations of ZK-RILs map.

### 2.4 Statistical analysis and QTL mapping

Statistical analysis was conducted using the set of predicted genotype means (Best Linear Unbiased Predictors, BLUP) for six traits in four environments and GenStat 19th software (VSN International, Hemel Hempstead, United Kingdom). Pearson correlation was used to analyse the relationship among the traits. A total of five datasets, designated 17CD, 17DY, 18CD, 18DY and BLUP data (B), were used for further analysis.

Two softwares, QTLNetwork 2.1 (http://ibi.zju.edu.cn/index.html/BCL/software/qtlnetwork.html) based on the mixed-model composite interval mapping (MCIM) model and IciMapping 4.1 (http://www.isbreeding.net/) based on the inclusive composite interval mapping (ICIM) model, were both employed to detect additive and epistatic QTLs. By using QTLNetwork v2.1, one- and two-dimensional genome scans for QTLs were performed using a 10 cM testing window, a 0.1 cM walk speed and a 0.5 cM filtration window size. For QTL analysis with IciMapping 4.1, the threshold of LOD of 3.42 was used to declare the presence of a putative QTL based on the 1,000 permutation tests (*p* = 0.05) for each trait. The walking speed chosen for all QTLs was 1.0 cM, and the *p*-value inclusion threshold was 0.001.

In this study, a QTL with a phenotypic variance contribution (PVE) > 10% (on average) detected by either approach (ICMI or MCIM) was defined as a major QTL; a QTL repeatedly detected by both softwares was defined as a stable QTL.

### 2.5 Development of kompetitive allele-specific PCR markers

On the basis of the preliminary QTL mapping results, SNPs adjacent to the target intervals of the major QTLs were converted into KASP markers (Supplementary Table S1) following the previously described method (Li et al., 2021) and used to trace the targeted QTL.

## 3 Results

### 3.1 Linkage map construction

The ZK-RILs were genotyped using the Wheat55K SNP array, which yielded 13,651 polymorphic markers for the linkage analysis. After removing unlinked markers and SNPs with more than 10% missing data or a segregation distortion test *p* = 0.01, a unique high-density genomic map with 11,455 markers and a length of 2,155.72 cM was created (Supplementary Figure S1; Table 1; Supplementary Table S2). To represent each bin on the created map, 1,459 bin markers with the lowest miss rate in each bin were picked (Supplementary Figure S1). The A, B, and D genomes had map lengths of 652.15, 606.41 and 857.16 cM, respectively, and 4,396, 4,288 and 2,782 markers, respectively, resulting in a similar number of bins (495 for the A genome, 469 for the B genome and 495 for the D genome) (Table 1; Supplementary Table S2). The 11,455 markers were distributed unevenly over the 21 chromosomes, with 59 markers on 4D and 1,325 on 2A. On chromosomes 2A, 2B, 3B, 4A, 5D and 7A, six gaps (more than 20 cM but less than 30 cM) were observed (Supplementary Figure S1; Supplementary Table S2). Furthermore, chromosomes 1D (1D1, 1D2, and 1D3), 3A (3A1, 3A2, and 3A3), and 6D (6D1, 6D2 and 6D3) were classified into three groups based on their linkage (Supplementary Figure S1; Table 1; Supplementary Table S2). The number of markers in each bin ranged from 1 to 980, whereas the number of chromosomes in each bin ranged from 30 (4D) to 105 (5B) (Supplementary Table S2).

### 3.2 Comparative genomic analysis

Overall, 10,384 (90.66%) of the 11,455 mapped probes were best matches to 9818 CS contigs, with an average of 1.06 polymorphic markers per contig (Additional file 1). The SNP arrangement in the present genetic map matched that of the wheat genome assembly (Supplementary Figure S2). In addition, 90.86% of the 11,455 mapped SNPs exhibited consistency with their physical position, whereas 5.85% were mapped to the genetic map for their homologous chromosomes, 0.72 percent were localized to different chromosomes within the same subgenome, and 2.57% were disordered (Supplementary Tables S2, S3). Furthermore, 3.04% (348 SNPs), 74.36% (8,517 SNPs), and 13.39% (1,534 SNPs) of the SNP markers were classified as cSNPs, iSNPs, and pSNPs, respectively (Additional file 1).

According to contig information, 4,257 markers in the ZK-RILs map shared contigs with 3,832 markers and 766 markers in previously reported genetic maps based on the Wheat 660K (Cui et al., 2017) and 90K SNP arrays (Wang et al., 2014), respectively (Supplementary Figure S3). Only 343 markers on 19 chromosomes (excluding 1B and 3B) shared contigs with three different arrays (Supplementary Figure S3, Additional file 2). These markers and the physical information associated with them could be used to create integrated genetic maps as well as to compare locus results from different chips.

**FIGURE 1:**
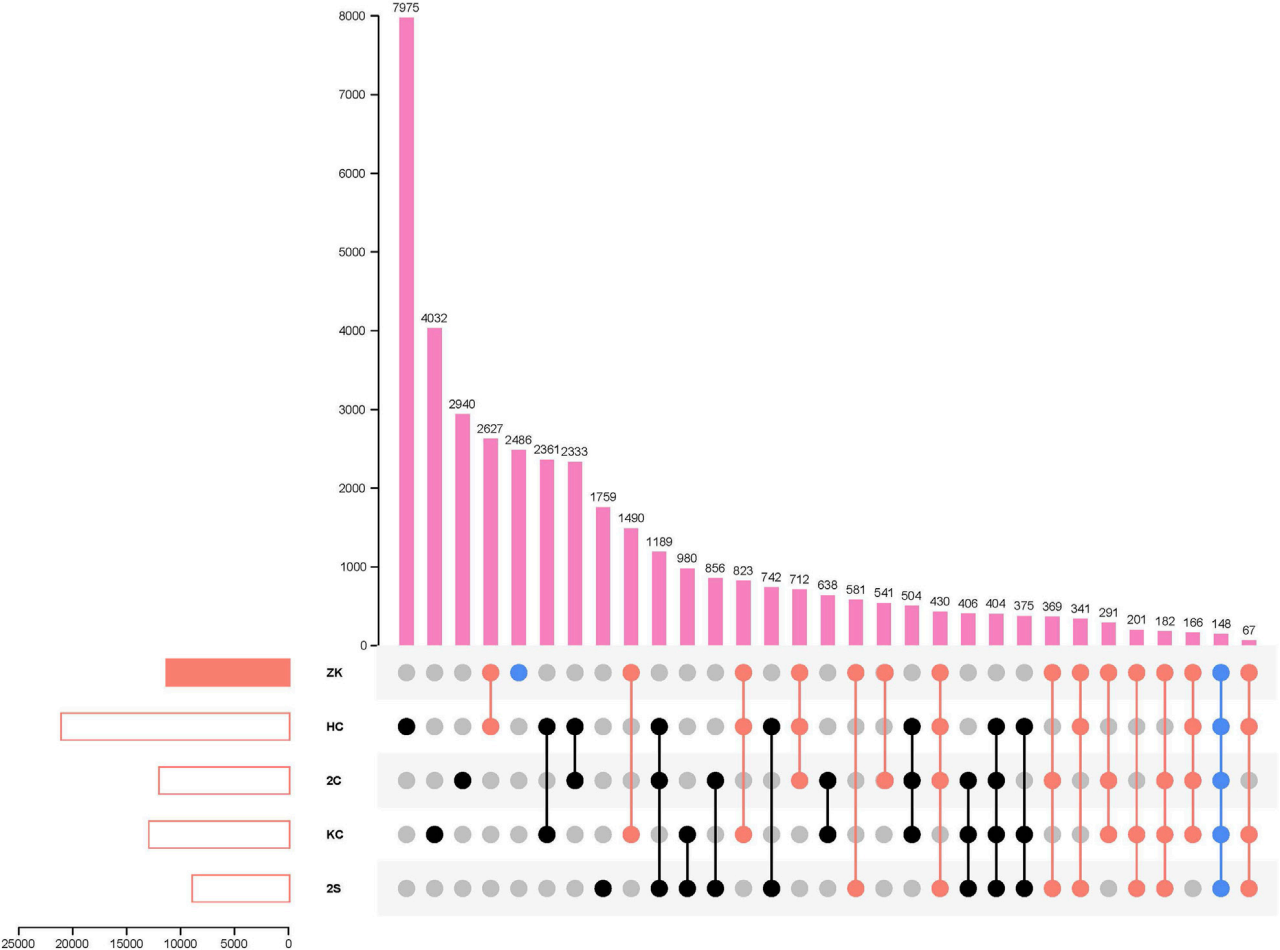
Venn diagram of the constructed Wheat 55K SNP array-derived genetic map based on ZK-RILs and the other four Wheat 55K SNP array-derived genetic maps for different populations.

Furthermore, when compared to the other 55 K SNP array-derived genetic maps for hexaploid wheat (Liu et al., 2018; Liu et al., 2020; Li et al., 2021; Lin et al., 2021), a total of 8,970 common markers were identified in these four maps: 2C-RILs map (Liu et al., 2018), 2S-RILs map (Liu et al., 2020), KC-DHs map (Li et al., 2021) and HC-RILs (Lin et al., 2021) (Figure 1; Supplementary Table S4). The most identical markers (2,627 SNPs) were discovered between ZK-RILs and HC-RILs maps (Lin et al., 2021), while only 541 common markers were discovered between ZK-RILs and 2C-RILs maps (Liu et al., 2018), which may be related to the number of markers in their respective genetic maps and the mapping parents’ kinship. In particular, 2,486 special markers for ZK-RILs were likely less reported, showing that the development of new genetic populations and genetic maps could aid in the discovery of novel genes or alleles. Across five maps, just 148 common markers were detected. These shared markers facilitated the mapping of gene loci and pan-genomic studies. This small number of shared markers underlines the need for distinct genetic maps from different genetic backgrounds to expand and update wheat genetic and genomic information.

### 3.3 Phenotypic evaluation and correlation analysis

**FIGURE 2:**
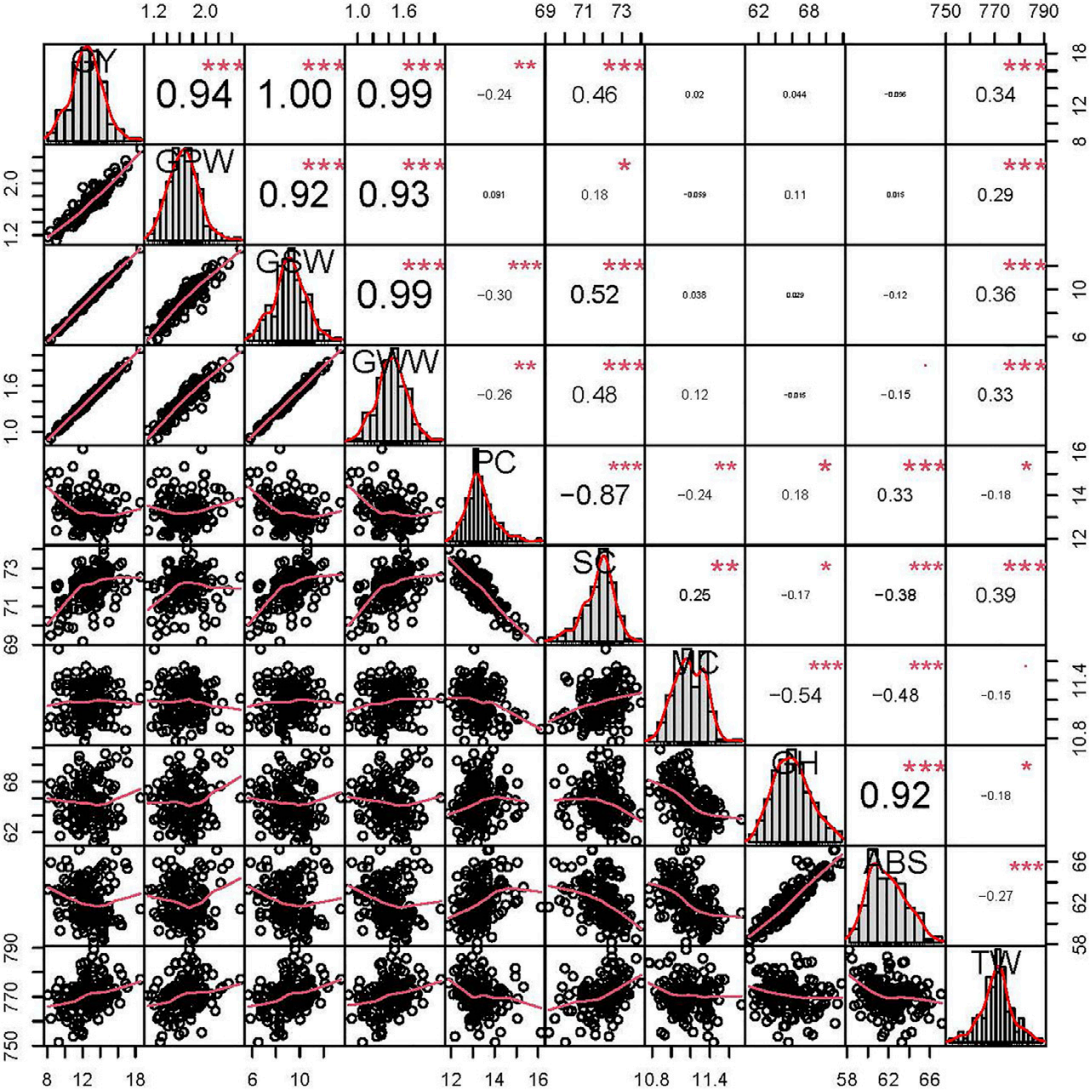
Phenotypic performance, distribution, and correlation coefficients of grain yield (GY), grain protein weight per plant (GPW), grain starch weight per plant (GSW), grain water weight per plant (GWW), protein content (PC), starch content (SC), moisture content (MC), grain hardness (GH), water absorption (ABS) and test weight (TW) in the ZK-RILs based on the BLUP data. “*”, “**” and “***” represent significance at *p* < 0.05, *p* < 0.01, and *p* < 0.001, respectively.

In all environments, ZKM138 clearly outperformed KCM2 in grain yield and its nutrient compositions (GPW, GSW and GWW), and ZKM138 had a relatively higher SC but a lower PC than KCM2 (Supplementary Table S5). In terms of GRRTs, ZKM138 only had significantly (*p* < 0.05) stronger GH and ABS but relatively lower TW than KCM2. The measured traits in the ZK-RILs showed transgressive segregation (Supplementary Table S5) and approximately continuous variance (Figure 2). Heritability ranged from 38.95% (ABS) to 89.46% (MC). The ANOVA results revealed that the genotype variance and the environmental effects of the investigated traits were both significant at *p* < 0.001 (Supplementary Table S5).

All correlation coefficients (r) (Figure 2) were considerably positive between GY and its three compositions (r > 0.92), including the correlation coefficient of GPW-GSW. However, PC was significantly negatively correlated with SC (r = −0.87), showing their well-known trade-offs. Except for the substantially strong correlation coefficient between ABS and GH (r = 0.92), significantly negative correlation coefficients (r = −0.18/−0.27) were observed for TW-GH and TW-ABS, respectively. In addition, for all measured traits, only SC and TW were shown to be significantly correlated with all of the other traits, particularly positively correlated with all four grain yield related traits, which were crucial determinants for wheat comprehensive commerciality.

### 3.4 Additive QTL mapping

#### 3.4.1 Additive effect analysis in combined datasets based on the MCIM model

Using QTLNetwork 2.1, a total of 62 additive QTLs for ten measured traits were identified, distributed on 18 chromosomes except 1B, 1D, and 6B, and accounting for 0.25%–34.39% of the phenotypic variation (Supplementary Figure S4; Table 2; Supplementary Table S6). Seven and seventeen QTLs were classified as the major (PVE>10%) and moderate (5% < PVE<10%) QTLs, respectively, while the remaining loci were classified as the minor QTLs (PVE<5%).

In detail, for PC, SC, and MC, one major QTL for PC (*QPc.cib-4D*) was discovered on chromosome 4D, accounting for 10.49% of the phenotypic variation, and colocated with a moderate QTL for SC, *QSc.cib-4D.1*. The ZKM138-derived alleles decreased PC but increased SC at this locus, possibly providing the genetic foundation for the PC-SC trade-offs (Supplementary Figure S4; Table 2; Supplementary Table S6). Another major MC-related QTL, *QMc.cib-5D.1*, was discovered on chromosome 5D, with a ZKM138-derived allele decreasing MC.

For grain grading related traits, two major QTLs, affecting GH (*QGh.cib-5D.1*) and ABS (*QAbs.cib-5D.1*) explained 30.67%–34.39% of the phenotypic variance and were all mapped near *Pina* on chromosome 5D (Supplementary Figure S4; Table 2, Supplementary Table S6). At this location, a ZKM138-derived allele may enhance both GH and ABS. Three moderate QTLs were discovered on chromosomes 2A (*QTw.cib-2A*), 6D2 (*QTw.cib-6D2*), and 7D (*QTw.cib-7D*), respectively. Among them, *QTw.cib-6D2* colocalized with another moderate QTL for MC (*QMc.cib-6D2*), with ZKM138-derived alleles decreasing TW but increasing MC.

Thirty-one QTLs were discovered for grain yield and three physical compositions (7 for GY, 9 for GWW, 8 for GPW, and 7 for GSW) (Supplementary Figure S4; Table 2; Supplementary Table S6). The interval *Rht2*–A*X-108905056* on the 4D chromosome was identified, which simultaneously clustered three major QTLs for GY, GPW, and GSW (*QGy.cib-4D*, *QGpw.cib-4D*, and *QGsw.cib-4D*) and one moderate QTL for GWW (*Gww.cib-4D*). The ZKM138 allele improved all four traits at this locus. Furthermore, the colocated interval on chromosome 2B grouped four QTLs (*QGy.cib-2B.1*, *QGsw.cib-2B.1*, *QGpw.cib-2B.1*, and *QGww.cib-2B*) with a moderate influence on all four traits and a positive additive effect from KCM2.

#### 3.4.2 Additive effect analysis in single dataset based on the ICIM model

Using IciMapping 4.1, twenty QTLs with LOD thresholds greater than 3.42 were identified (Supplementary Figure S5; Table 2; Supplementary Table S7). These QTLs explained 5.46%–35.56% of the phenotypic variation, with an average LOD value ranging from 3.47 to 12.06. Among these loci, fourteen were consistently significant under both test conditions, eight of which clustered in three intervals on chromosomes 4D (*QGy.cib-4D*, *QGpw.cib-4D* and *QGww.cib-4D.1*), 5D (*QAbs.cib-5D.1*, *QGh.cib-5D.1* and *QMc.cib-5D.1*) and 6D (*QMc.cib-6D2* and *QTw.cib-6D2*), respectively. Noticeably, all three involved QTLs on chromosome 5D presented relatively higher PVE (35.56%, 32.98% and 24.46%, respectively) based on the ICIM model, which was consistent with the results detected by QTLNetwork 2.1, and they were all expressed across all datasets and thus were classified as major stable QTLs. Furthermore, because two loci on the 6D2 linkage (*QMc.cib-6D2* and *QTw.cib-6D2*) were persistently significant in all environments and repeatedly detected by two approaches, these two QTLs were modest but stable QTLs simultaneously controlling MC and TW. Only the GY composition-related QTLs were clustered for the interval on chromosome 4D.

### 3.5 Epistatic QTL mapping

For QTLnetwork 2.1, 47 pairs of epistatic QTLs were found, with PVE ranging from 0.91 to 11.77% (Supplementary Figure S5; Table 3; Supplementary Table S8). Two pairs that generated epistatic interactions for MC (*QMc.cib-4D*/*QMc.cib-5D.1* and *QMc.cib-5B*/*QMc.cib-6A*) also demonstrated an additive effect. Another QTL, *QAbs.cib-5D.1*, was shown to have an epistatic interaction with another epistatic QTL that had no additive effect (*QAbs.cib-3D*). There was just one pair of digenic QTLs (*QGy.cib-4A.1*/*QGy.cib-4A.2*) that showed a significant epistatic interaction effect (>10%) that explained 11.77% of the phenotypic variation. Except for this pair, the other 46 epistatic QTL pairs all interacted epistatically across different chromosomes.

ICIMapping 4.1 discovered thirty-five pairs of epistatic QTLs involving only three measured characteristics, namely ABS (8 pairs), GH (25 pairs), and GPW (1 pair) (Supplementary Figure S6, Supplementary Table S9). The other seven traits showed no epistasis over the LOD threshold (5.62). Only the epistatic interaction between chromosomes 1B (*AX-94383682*–*AX-108841881*) and 5A (*AX-110368018*–*AX-109731422*) demonstrated a higher PVE for GPW of 11.53%, which was also significant by QTLNetwork 2.1. Unlike the epistatic QTL mapping results from QTLNetwork 2.1, only three QTL pairs interacting across different chromosomes were detected by ICIMapping 4.1, but the epistatic effect of the remaining 32 pairs (91.43%) was generated by loci on the same chromosome.

### 3.6 QTL clusters

**FIGURE 3:**
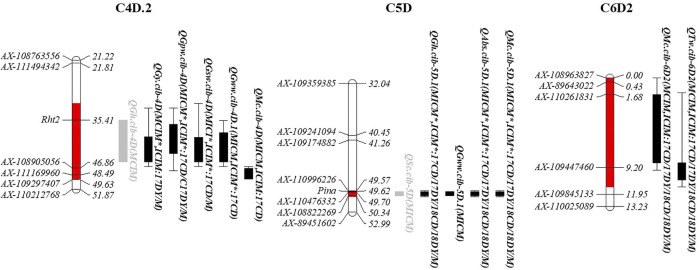
Genomic intervals harbouring at least two stable QTLs. The red interval on chromosomes indicates the confidence interval of this cluster. The rectangles to the right of the chromosome represent the QTLs. Black indicates significance under both detection approaches and is a stable QTL, while grey indicates it is only significant by one approach. The bar represents the confidence interval detected in one software, and the rectangle represents the overlapping confidence intervals. The brackets after the QTL name follow the detection method, and the asterisk means that both additive and epistatic effects are detected.

In this study, 13 intervals clustering two or more additive QTLs were observed (Supplementary Table S10). Only C4D.2, C5D, and C6D2 had at least two stable QTLs that could be detected repeatedly using both MCIM and ICIM methods (Figure 3; Supplementary Table S10). C4D.2, involving the famous semidwarfing gene *Rht2*, was clustered by four QTLs affecting grain yield and compositions with higher PVE values (moderate or major), one minor QTL for GH, and one moderate QTL for MC, showing that it mostly affects total yield and nutrient yield. C5D had two major QTLs for GH and ABS, probably associated with the famous hardness locus, *Pina*; two major or moderate QTLs for SC and MC; and one minor QTL for GWW, indicating its potential role in milling and processing. Furthermore, C6D2 has two moderate but consistent QTLs for TW and MC (*QTw.cib-6D2* and *QMc.cib-6D2*), implying a relation to wheat grading.

**FIGURE 4:**
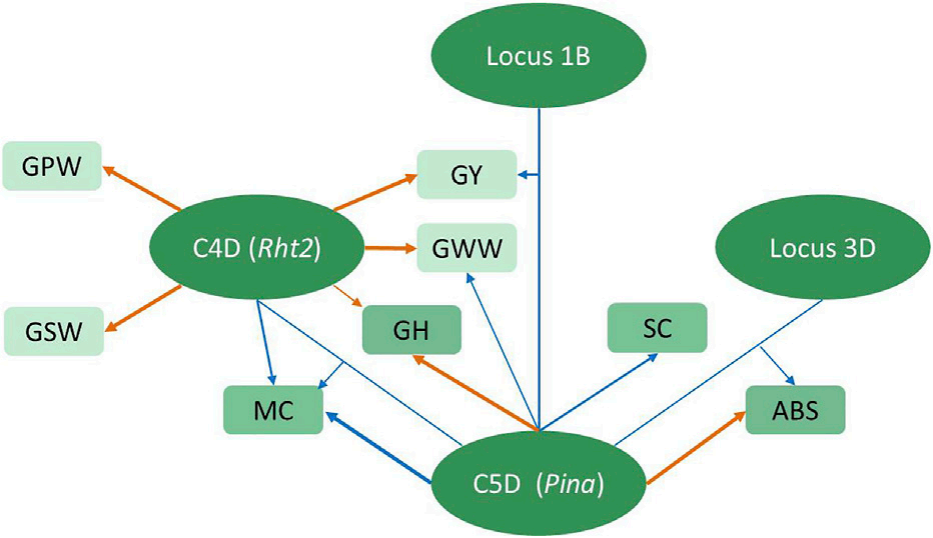
Schematic representation of the regulation of C4D and C5D on related traits. The ellipses represent loci, where C4D represents the *Rht2*–*AX-109297407* segment on chromosome 4D, possibly controlled by *Rht2*, C5D represents the *Pina*–*AX-110476332* segment on chromosome 5D, possibly controlled by *Pina,* Locus 1B represents the *AX-111234844*–*AX-110918909* segment on chromosome 1B, and Locus 3D represents the *AX-94528475*–*AX-110443023* segment on chromosome 3D. Rectangles represent the corresponding traits. Arrows emanating directly from the locus to the trait represent additive regulatory effects of the locus on the corresponding trait. Arrows linking two loci to the trait represent epistatic interactions between two loci regulating the corresponding trait. The different arrow thicknesses represent the magnitude of the PVE values. Orange arrows represent positive alleles derived from Zhongkemai138, and blue arrows represent positive alleles derived from KCM2.

In addition to the additive QTLs, twenty-three epistatic QTLs controlling various attributes showed colocation in 10 intervals (Supplementary Table S11), indicating that epistasis could be pleotropic or linked. The epistatic interaction generated by the *AX-109412207–AX-108965184* interval on chromosome 3B and the *AX-109200636–AX-110438066* interval on chromosome 6D2 was shown to simultaneously influence GPW and GSW. Notably, *Pina* involved the epistatic interaction for GY, ABS, and MC with three different intervals on chromosomes 1B, 3D, and 4D (Figure 4), as well as the main additive effect on ABS, MC, GH, SC, and GWW. Among them, its interaction involving the MC was found to be involved with *Rht2*, which had the main effect on GY and compositions, indicating the complexity of the genetic effect on grain characteristics between these two genes, which might be explained by crucial structural genes being controlled by different regulatory factors to affect different traits.

### 3.7 Effects of *QTw/Mc.cib-6D2* on grain performance in the ZK-RILs

**FIGURE 5:**
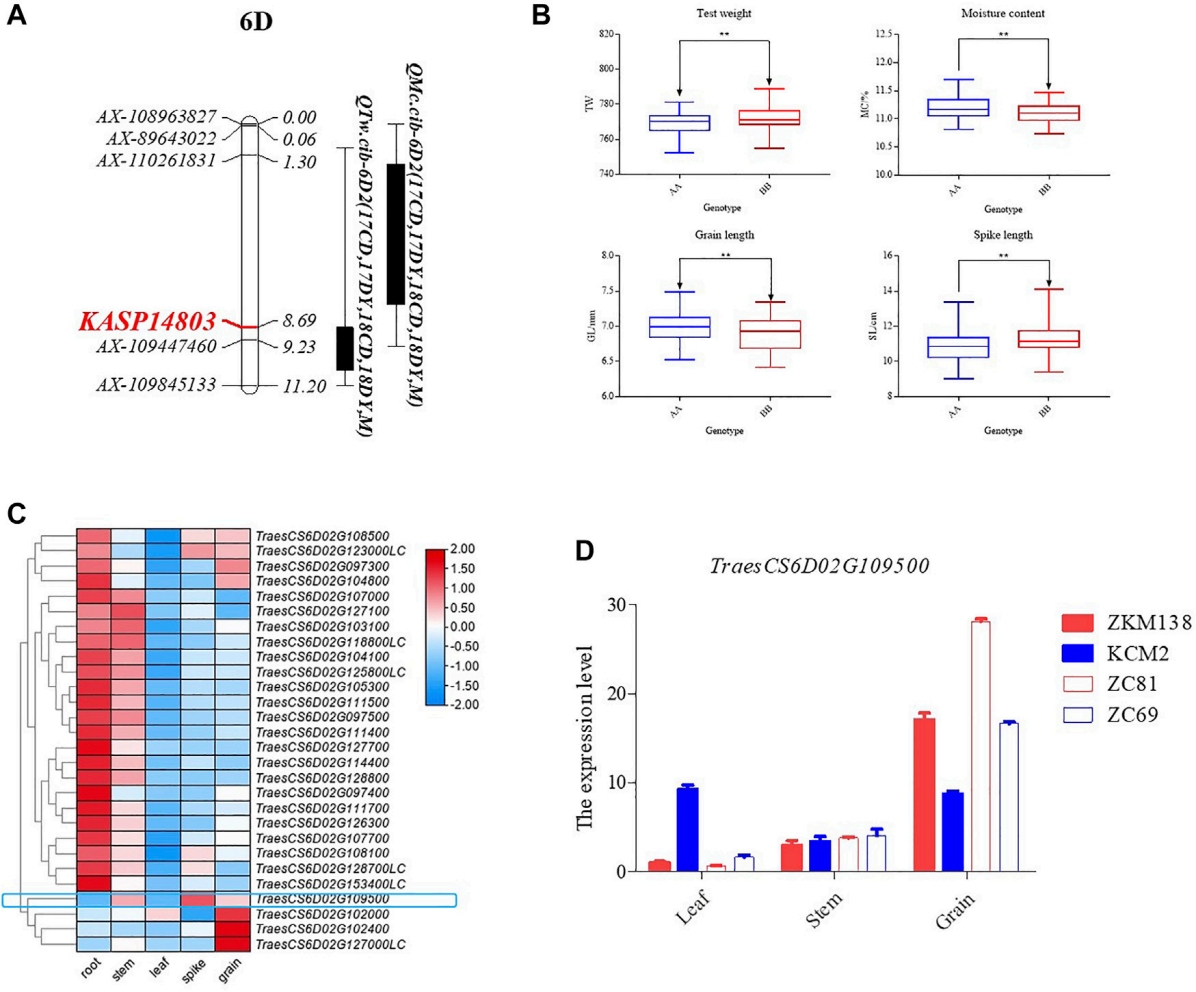
The candidate interval for *QTw/Mc-6D.2*. **(A)** The developed KASP marker for detecting *QTw/Mc-6D.2*s. The marker in red indicates the flanking KASP marker for *QTw/Mc-6D.2*. **(B)** The effect of *QTw/Mc-6D.2* on the corresponding traits validated by *KASP14803*. **(C)** Heatmap of the expression profiles for the candidate genes in this interval. **(D)** qPCR validation for *TraesCS6D02G109500*.

To validate the loci on chromosome 6D, a total of 15 KASP markers were developed and subsequently integrated into the genetic map of 6D2 linkage to trace *QTw.cib-6D2* and *QMc.cib-6D2* (Supplementary Table S1; Supplementary Table S12). Finally, a new genetic map for 6D2 linage with 51 markers were constructed to QTL mapping for the target phenotypes, i.e., TW and MC. Using the updated map, the confidence interval (CI) of *QTw.cib-6D2* and *QMc.cib-6D2* was relocated around a flanking KASP marker, *KASP14803* (Figure 5A) and its original flanking marker *AX-109447460*. Using the flanking marker *KASP14803*, ZK-RILs were genotyped to examine whether their accompanying phenotypes could be distinguished. Finally, TW and MC were remarkably different when the genotype was the AA (ZKM138 genotype) or BB (KCM2 genotype) (Figure 5B). Aside from TW and MC, the group with ZKM138-derived alleles exhibited significantly shorter spike length (SL) but longer kernel length (KL) than the group with KCM2-derived alleles at this locus (Figure 5B). This result indicated that this locus not only controlled grain features but also affected spike formation and suggested that *KASP14803* was tightly linked to this target locus and could be convenient for the further molecular breeding.

## 4 Discussion

According to the physical location of these mapped SNPs in this study, these loci cover the majority of the wheat genome, demonstrating their applicability for further candidate gene excavation. However, several regions still failed to fill with markers on chromosomes 1D, 3A, and 6D, which were all composed of three individual linkage groups in ZK-RILs (Supplementary Figure S1; Supplementary Table S2), possibly explained by the low recombination events and frequency polymorphism in the corresponding intervals (Cui et al., 2017; Liu et al., 2018; Ren et al., 2018). Interval inversions were also found on multiple chromosomes, including 4D, 5D, and 7A (Supplementary Figure S1; Supplementary Table S2). These breakages and inversions are also frequently detected in other genetic maps (Cui et al., 2017; Liu et al., 2018; Ren et al., 2018; Li et al., 2021).

Furthermore, we discovered that in the other 55K SNP array derived genetic maps, some of their genetic markers do not match the physical location but share a similar position to the ZK-RILs genetic map (Supplementary Table S4), indicating that physical positions are relative and that different genetic backgrounds could induce alterations in genome arrangement information. The physical position of *AX-111180568*, for example, was on the 2D chromosome, but it was localized to chromosome 2A in this ZK-RILs map and the other four 55K array generated maps (Liu et al., 2018; Liu et al., 2020; Li et al., 2021; Lin et al., 2021) (Supplementary Table S4). Furthermore, the 6D genetic map is generally consistent with the physical map and other genetic maps, indicating that it might be well conserved. It should also be highlighted that some markers specific to the ZK-RILs genetic map were not consistent with physical positions and differed from other genetic map locations. For example, seventeen SNP markers mapped in the 31.06 cM-bin on chromosome 6D of the ZK-RILs genetic map were from chromosome 1A according to both physical location and other genetic maps (Cui et al., 2017; Lin et al., 2021) (Supplementary Table S2; Supplementary Table S4). This result demonstrated that maps from different genetic backgrounds could reflect their own genetic features inherited from different parents, laying the foundation for novel gene mining.

The dwarfing genes and hardness loci are commonly distributed in common wheat and affect wheat yield and quality (Ellis et al., 2002; Li et al., 2006; Qamar et al., 2014). To validate the usability of this map, we added molecular markers of the dwarfing gene *Rht2* (Ellis et al., 2002) and hardness locus *Pina* (Giroux and Morris, 1998; Li et al., 2006) for genotyping ZK-RILs and finally found that, as expected, they were integrated on chromosomes 4D (Börner et al., 1996) and 5D (Giroux and Morris 1998) and successfully located major QTLs controlling plant height (data not shown) and grain hardness (*QGh.cib-5D.1*), respectively. In this study, four major QTLs for grain yield and its physical composition were clustered at this Rht2 locus. Consistent with previous research indicating that *Rht2* has an effect on yield performance (Borrell et al., 1991), ZKM138-derived alleles at this locus could improve yield, possibly offering the genetic basis for ZKM138s excellent yield performance. Despite the fact that all three seed compositions, GWW, GSW, and GPW, exhibited a significant and positive correlation with grain yield (Figure 2), not all QTL influencing composition weight were able to influence the final grain yield. In contrast, only four of nine QTLs for GWW co-localized with GY-QTL (Supplementary Tables S6, S7). However, in the C4D.2 cluster associated with *Rht2*, not only GY- but also all GWW-, GSW-, and GPW-related QTLs were colocated in this region, indicating that *Rht2* may enhance the accumulation of all these physical substances to increase final grain yield, which might be related to the different competition capacity for carbohydrates or proteins between stem elongation and grain development (Gent and Kiyomoto, 1997; Triboi and Triboi-Blondel, 2002; Shearman et al., 2005). Although *Rht2* raised GY and its major compositions, there was no significant additive effect on the concentration parameters of protein and starch (PC and SC). Only an additive effect of *Rht2* controlling MC (*QMc.cib-4D*) was detected and showed an epistatic interaction for MC with *Pina* (Figure 4), indicating the existence of a complex regulatory network between these two genes and the potential relationship between plant height, yield composition, and grain hardness might be involved with the water accumulation and metabolism during grain development.

On the other hand, the C5D mapped additive QTL for SC, consistent with previous studies reporting that *Pina* regulating the grain starch granule synthesis (Capparelli et al., 2003; Wanjugi et al., 2007), indicating *Pina* is one of the dominant genes influencing flour quality. Its direct additive effect on GY has rarely been reported and also was not detected in this study. However, the epistatic interaction on GY between *Pina* and the 589.95–668.67 Mb interval on chromosome 1B (*QGy.cib-1B*) was noticed. Therefore, the contribution of *Pina* to yield formation might also involve the accumulation of water and be governed by the epistasis of this *QGy.cib-1B*, taking into account the additive effect of GWW (*QGww.cib-5D*).

Previous research has shown that the Wheat 55K SNP array can satisfy primary QTL mapping in a genetic population of a similar size to that of this study (Liu et al., 2018; Ren et al., 2018). QTL mapping utilizing the primary mapping population to appropriately identify and find the genes responsible for particular agronomic traits is used for both major and moderate/minor QTLs (Cui et al., 2017). Previous research has shown that cloned genes are close to the positions detected in their primary mapping population (Cui et al., 2017). Three clusters on chromosomes 4D, 5D, and 6D were highlighted in this investigation because they all had at least two additive stable major/moderate QTLs that could be discovered repeatedly by both QTLNetwork 2.1 and IciMapping 4.1 (Table 2; Supplementary Table S10). However, C4D.2 and C5D have been discovered to be controlled by known genes. C6D2 for MC and TW might harbour novel loci, probably important in seeds storage and grading. According to the comparison of the resequencing results (data not shown) between the two parents, there were 174 identified genes with SNP or Indel variation (excluding intergenic regions) throughout the entire possible interval (60–95 Mb of the 6D chromosome). According to the public expression database (http://202.194.139.32/expression/wheat.html), only 28 of them had an overall expression level of greater than 5 in all tissues (Figure 5C). Only three genes were identified to be moderately highly expressed concurrently in grain and spike: *TraesCS6D02G123000LC*, *TraesCS6D02G108500*, and *TraesCS6D02G109500* (Figure 5C). Among them, *TraesCS6D02G109500*, which is located near 75 Mb of the 6D chromosome and encodes an aleurone layer morphogenesis protein, which might affect water adsorption and loss properties through controlling the development of the aleurone layer (constitutive of grain bran tissue) (Ying et al., 2020), finally regulating the water concentration and other related traits in seeds, was more likely the possible candidate gene, given its functional annotation for grain features and closeness to the overlapping candidate interval, but this conclusion is still preliminary.

**TABLE 1:** Details of the ZK-RILs genetic map.

Chromosome	Group	Bin	Marker number	Length (cM)	Bin resolution (cM)	Marker density (cM)
chr1A	1	56	767	45.02	0.80	0.06
chr2A	1	71	1,325	123.26	1.74	0.09
chr3A	1	14	72	10.68	0.76	0.15
	2	39	229	23.43	0.60	0.10
	3	16	74	25.92	1.62	0.35
chr4A	1	60	223	119.41	1.99	0.54
chr5A	1	99	484	117.19	1.18	0.24
chr6A	1	65	791	84.41	1.30	0.11
chr7A	1	75	431	102.83	1.37	0.24
chr1B	1	76	484	78.61	1.03	0.16
chr2B	1	68	439	108.79	1.60	0.25
chr3B	1	40	424	81.03	2.03	0.19
chr4B	1	63	774	103.24	1.64	0.13
chr5B	1	105	811	65.02	0.62	0.08
chr6B	1	52	888	58.41	1.12	0.07
chr7B	2	65	457	111.33	1.71	0.24
chr1D	1	50	326	87.66	1.75	0.27
	2	10	110	37.23	3.72	0.34
	3	6	6	8.80	1.47	1.47
chr2D	1	84	637	164.43	1.96	0.26
chr3D	1	55	556	83.41	1.52	0.15
chr4D	1	30	59	85.44	1.45	1.45
chr5D	1	85	252	130.59	1.55	0.52
chr6D	1	17	65	32.44	1.91	0.50
	2	37	202	77.41	2.09	0.38
	3	16	62	11.02	0.69	0.18
chr7D	3	105	507	178.73	1.70	0.35
A genome	12	495	4,396	652.15	1.32	0.15
B genome	8	469	4,277	606.41	1.29	0.14
D genome	19	495	2,782	897.16	1.81	0.32
Total	27	1,459	11,455	2,155.72	1.48	0.19

Note: Density was calculated by dividing their added genetic length by their added bin markers.

**TABLE 2:** Stable additive QTLs detected by both the MCIM model and ICIM model.

Trait	QTL^a^	Marker interval	Range^b^	MCIM		ICIM	
				Additive effect	H^2^ (%)	Additive effect	PVE (%)
ABS	***QAbs.cib-5D.1*** *	*Pina–AX-110476332*	49.5–50.5	1.23	34.39	1.33	35.56
GH	***QGh.cib-5D.1***	*Pina–AX-110476332*	49.5–50.5	1.54	30.67	1.51	32.99
MC	QMc.cib-4D*	*AX-108905056–AX-111169960*	46.5–49.5	−0.05	7.29	−0.05	7.19
	***QMc.cib-5D.1***	*Pina–AX-110476332*	49.5–50.5	−0.08	19.79	−0.10	24.46
	*QMc.cib-6D2*	*AX-110261831–AX-109447460*	0–9.5	0.06	9.82	0.06	8.13
TW	*QTw.cib-6D2*	*AX-110261831–AX-109447460*	1.5–11.2	−1.93	7.81	−2.18	8.21
	*QTw.cib-7D*	*AX-110033966–AX-108785845*	29.5–34.5	−2.04	8.7	−2.25	8.68
GY	***QGy.cib-4D***	*Rht2–AX-108905056*	32.5–46.5	0.74	13.83	0.85	11.97
SC	*QSc.cib-2D*	*AX-109525831–AX-108960866*	40.5–43.3	0.17	3.45	0.30	8.96
GPW	*QGpw.cib-3B*	*AX-109819016–AX-110392622*	39.5–40.5	0.07	5.9	0.12	9.21
	*QGpw.cib-4B.1*	*AX-109294476–AX-111176263*	15.5–20.6	−0.08	7.59	−0.11	6.58
	***QGpw.cib-4D***	*Rht2–AX-108905056*	32.5–47.5	0.11	14.94	0.13	10.94
GSW	***QGsw.cib-4D***	*Rht2–AX-108905056*	31.5–46.5	0.51	12.28	0.62	11.78
GWW	***QGww.cib-4D.1***	*Rht2–AX-108905056*	35.5–46.5	0.06	8.4	0.10	11.79

a The QTL, in bold are major additive QTLs; *Indicates the additive QTLs, with epistatic effects.

b The overlapping interval identified by QTLNetwork 2.1 based on the MCIM, model and IciMapping 2.1 based on the ICIM, model.

**TABLE 3:** Stable epistatic QTLs detected by both the MCIM model and ICIM model.

QTL1	Marker interval 1	Range 1	QTL2	Marker interval 2	Range 2	MCIM		ICIM	
						AA	H^2^ (%)	AA	PVE (%)
*QGpw.cib-1B.1*	*AX-94383682–AX-108841881*	0.0–5.0	*QGpw.cib-5A*	*AX-109731422–AX-109342568*	94.9–97.0	0.03	1.51	0.09	11.53

The *TraesCS6D02G109500* expression pattern was preliminarily validated using qPCR, and the results were consistent with expectations, with grain expression being significantly higher than leaf and stem expression (Figure 5D). Furthermore, its expression was substantially higher in ZKM138 and line 81, both of which had ZKM138-derived genotypes validated by *KASP14803*, than in KCM2 and line 69, both of which had BB genotypes, indicating that the gene might have a role in grain features. This finding and the markers it yielded could be beneficial to grain performance genetic enhancement *via* MAS.

## 5 Conclusion

This study introduced a new genetic map using the Wheat 55K SNP array and presented a comparison with previously reported genetic and physical maps, which might provide information for wheat genetic and genomic studies. The additive and epistatic effects of QTLs were analysed using this map for six quality-related traits and four yield-related traits by two distinct QTL detection models. The major additive QTL affecting wheat grain yield and its compositions were localized around *Rht2*, suggesting that the dwarfing gene may affect yield by regulating the biomass accumulation of seed inner substances, while the major additive QTL influencing wheat seed hardness was localized around *Pina* and had epistatic interaction with *Rht2*. In addition to these two known genes, we also found a newly reported QTL for MC and TW on chromosome 6D that could be detected repeatedly in two different softwares, and finally located it around the flanking KASP marker (*KASP14803*). This marker could cleanly differentiate the MC and TW phenotypes separately and might be useful to the future molecular selection. Finally, a candidate gene encoding aleurone layer morphogenesis protein, *TraesCS6D02G109500*, was highlighted and requires further investigation and validation.

## Data Availability

The original contributions presented in the study are included in the article/Supplementary Material, further inquiries can be directed to the corresponding authors.
