# Supplementary material for: Construction of a novel Wheat 55 K SNP array-derived genetic map and its utilization in QTL mapping for grain yield and quality related traits
Source: Front Genet. 2022 Aug 26;13:978880. doi: 10.3389/fgene.2022.978880 (PMC9462458; doi:10.3389/fgene.2022.978880)
Supplement: Supplementary file 1 [file DataSheet1.zip › Supplemantary files/Supplemantary Figure S1-S6.pdf]

Construction of a novel Wheat 55 K SNP array-derived genetic map and its utilization in QTL mapping for grain yield and quality related traits

Xiaoli Fan [1], Xiaofeng Liu [1,3], Bo Feng [1], Qiang Zhou [1], Guangbing Deng [1], Hai Long [1], Jun Cao [4], Shaodan Guo [1], Guangsi Ji [1,3], Zhibin Xu [1*], Tao Wang [1,2*]

[*] *Corresponding Author.*

[*] xuzb@cib.ac.cn (Z. Xu), wangtao@cib.ac.cn (T. Wang).

1 Chengdu Institute of Biology, Chinese Academy of Sciences, Chengdu 610041, China

2 Innovative Academy for Seed Design, Chinese Academy of Sciences, Beijing 100101, China

3 University of Chinese Academy of Sciences, Beijing 100049, China

4 Yibin University, Yibin 644000, China

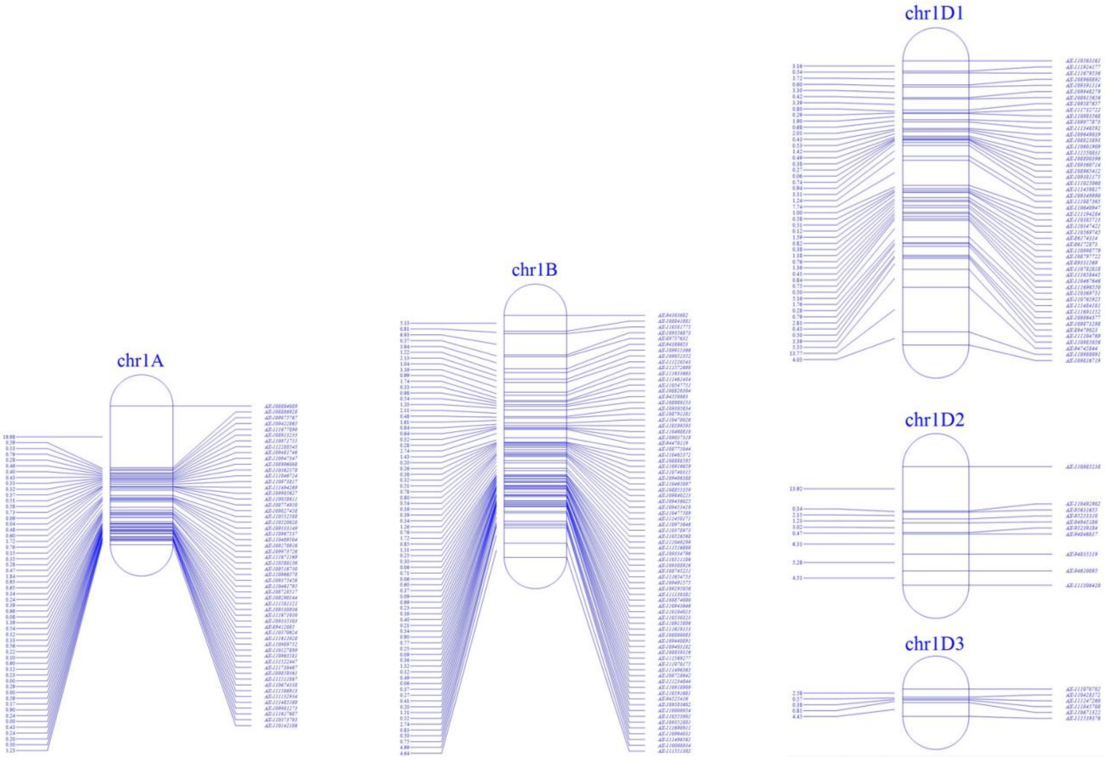

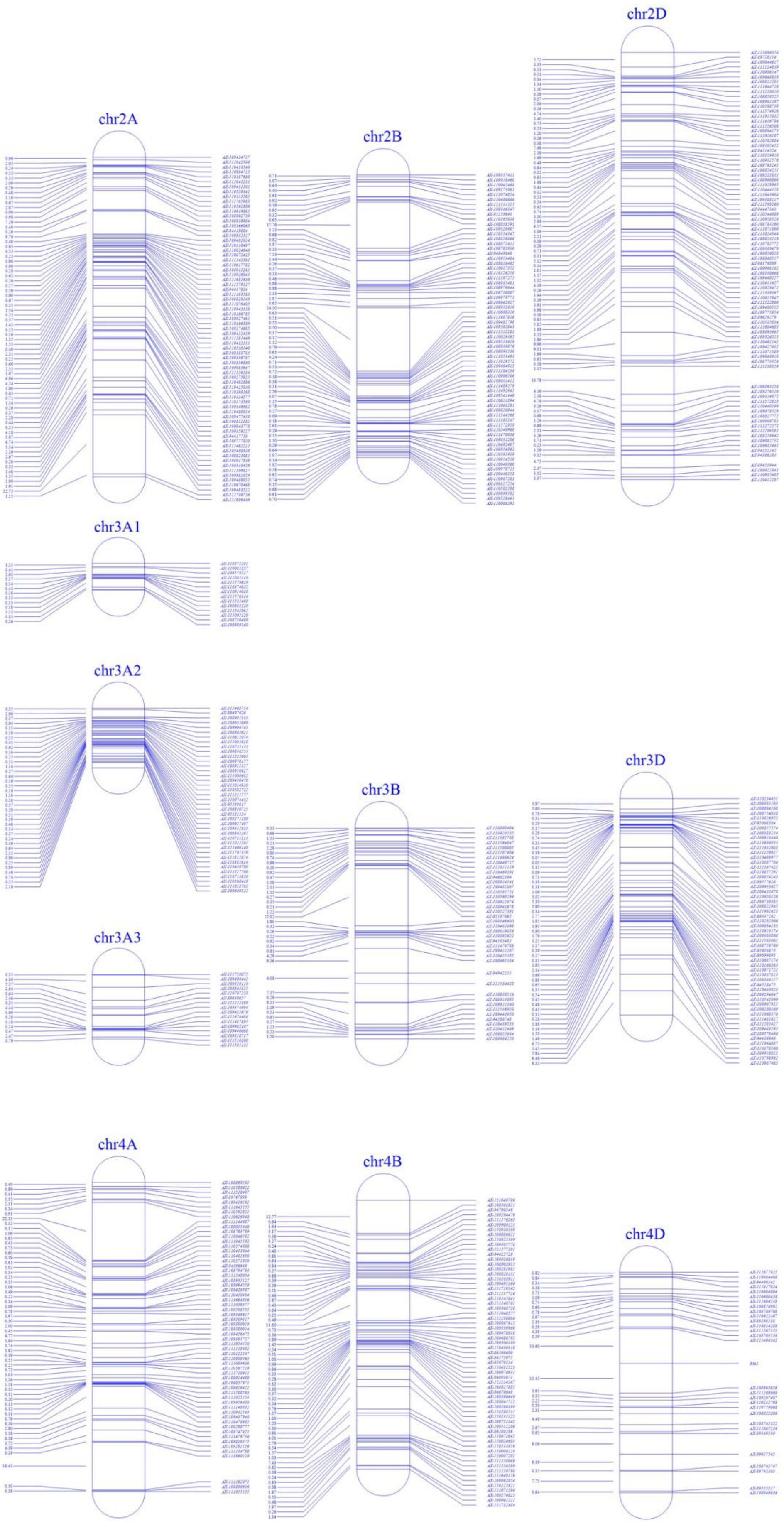

chr2A
chr2B
chr2D
chr3A1
chr3A2
chr3A3
chr3B
chr3D
chr4A
chr4B
chr4D

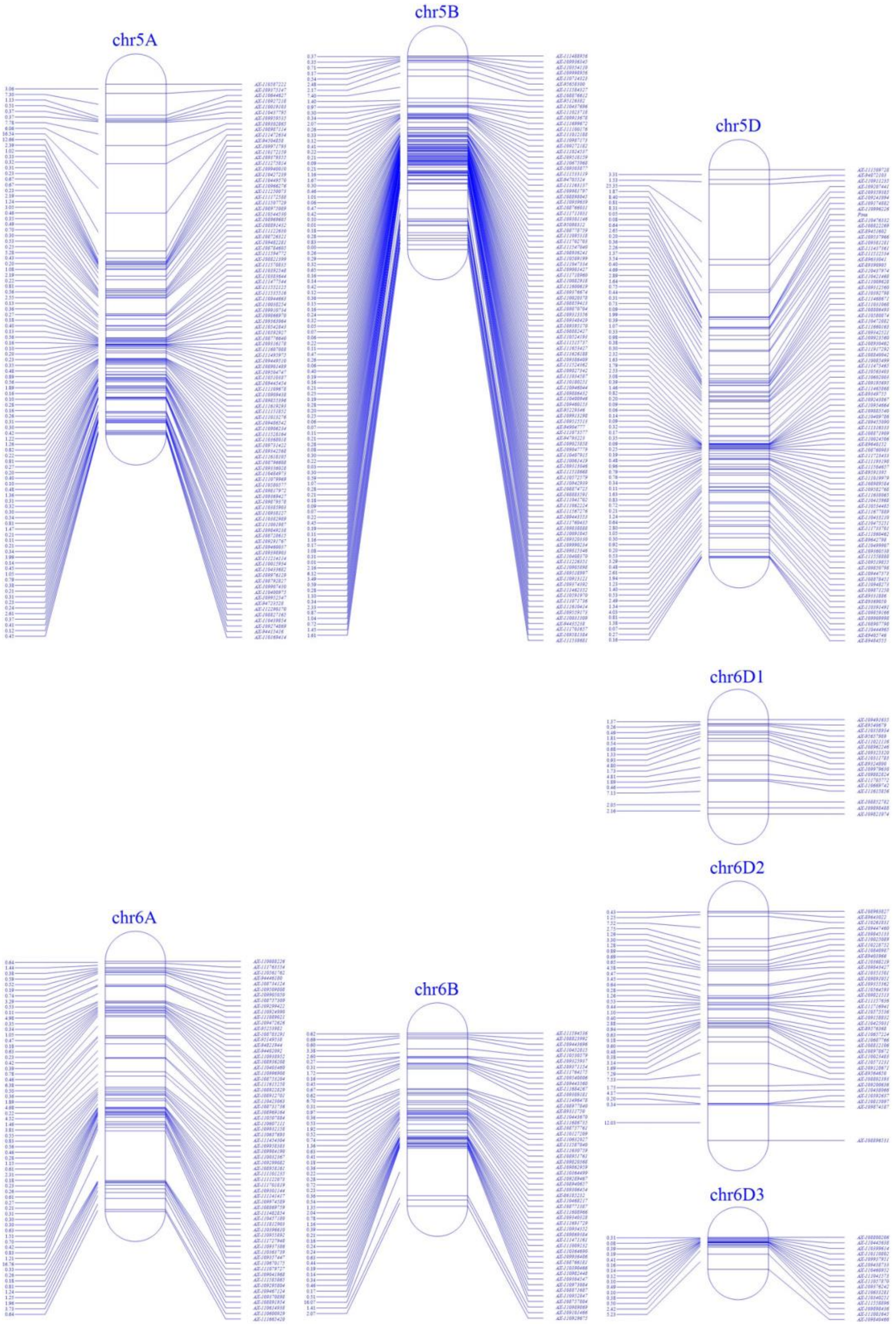
chr5A
chr5B
chr5D
chr6D1
chr6A
chr6B
chr6D2
chr6D3

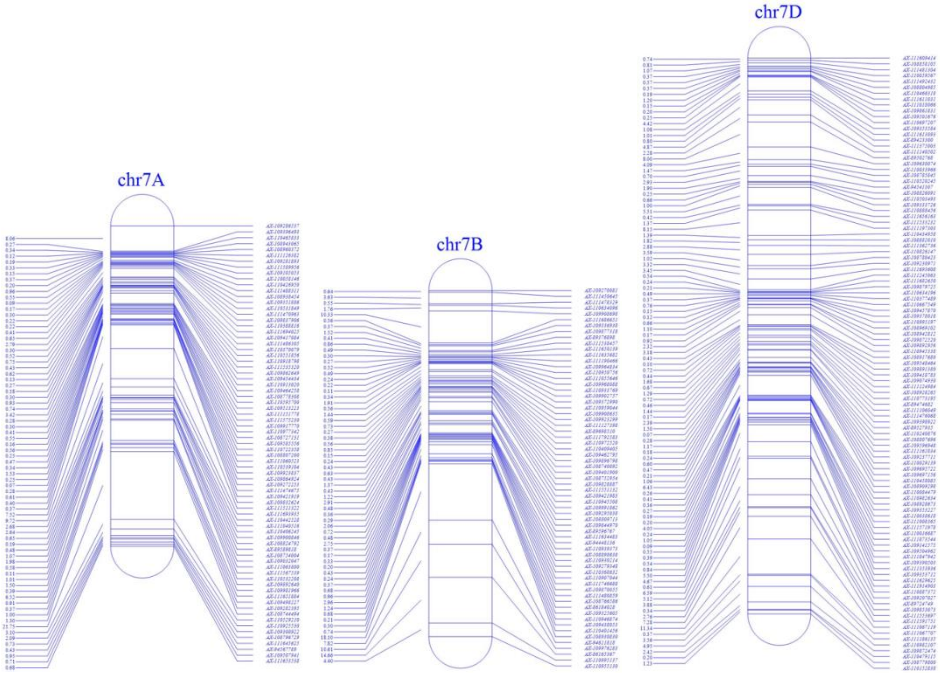

Figure S1 Genetic map of wheat developed using an RIL population derived from the cross of cultivars ZKM138 and KCM2. Short arms are at the top. For the redundant loci that showed co-segregation in the 152 ZK-RILs, only one unique informative bin marker is shown in this figure. The intervals and names of the marker loci are on the left and right side of each chromosome, respectively.

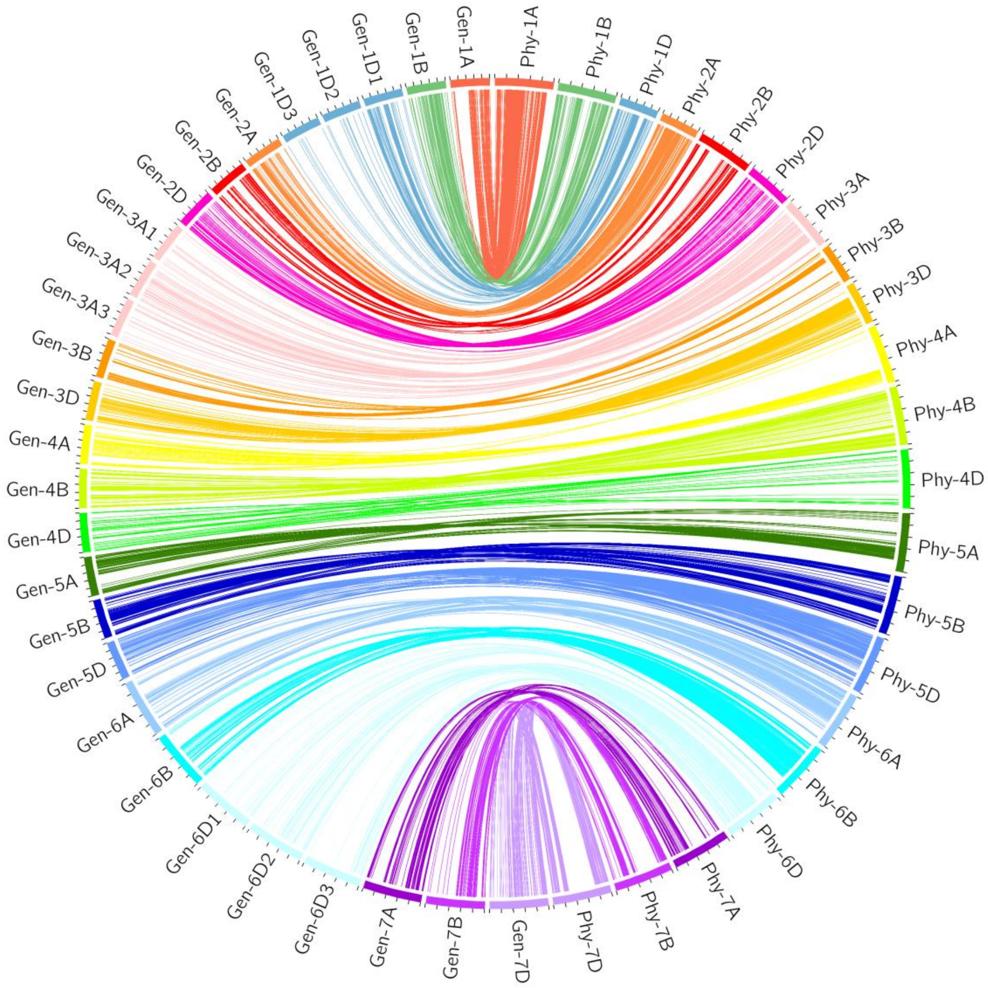

Figure S2 Schematic representation of the co-linearly relationships between each marker in the wheat genetic and physical maps. Gen-1A to Gen-7D represent the 21 wheat chromosomal genetic maps released in this paper; Phy-1A to Phy-7D represent the 21 wheat chromosomal physical maps, which were constructed by assigning 11455 SNPs to the wheat genome assembly using SNP flanking sequences as the query. For the redundant loci that showed co-segregation in the 152 ZK-RILs, only bin markers are shown in this figure.

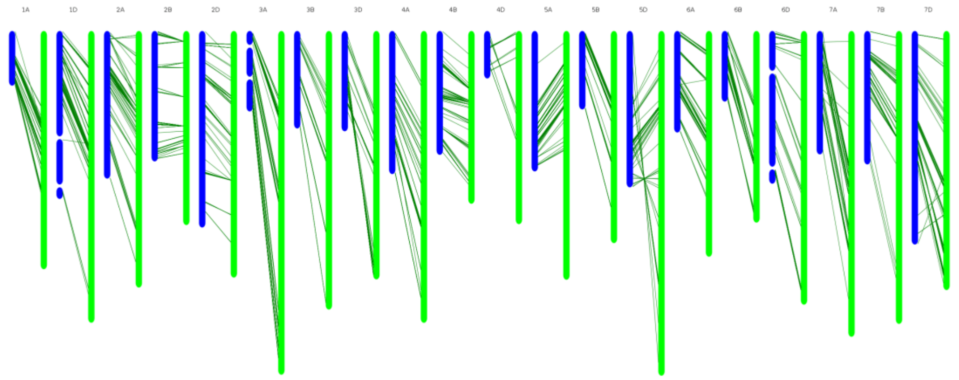

a

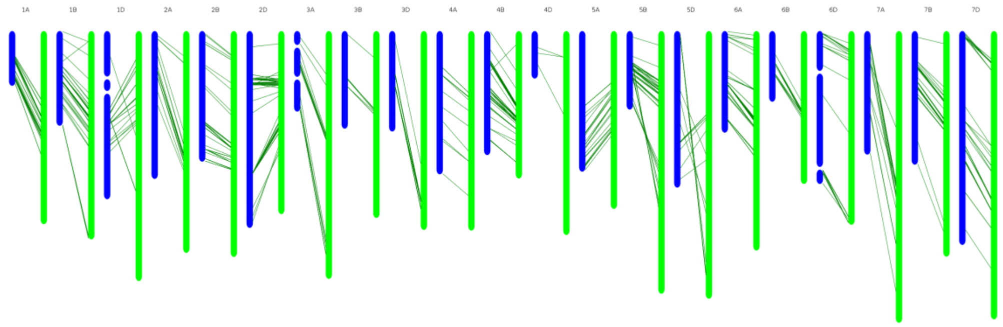

b

Figure S3 Comparison between the ZK-RILs wheat genetic maps based on the Wheat 55K SNP array and the genetic map based on the (a) Wheat 660K SNP array (Cui et al. 2017) and (b) Wheat 90K SNP arrays (Wang et al. 2016).

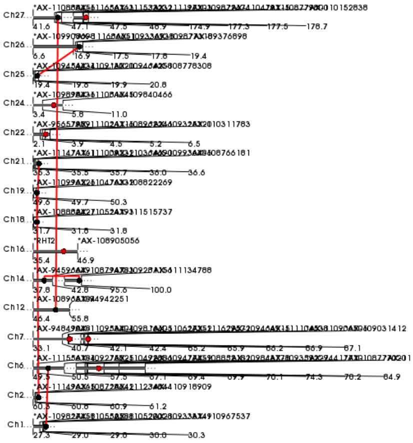

GY

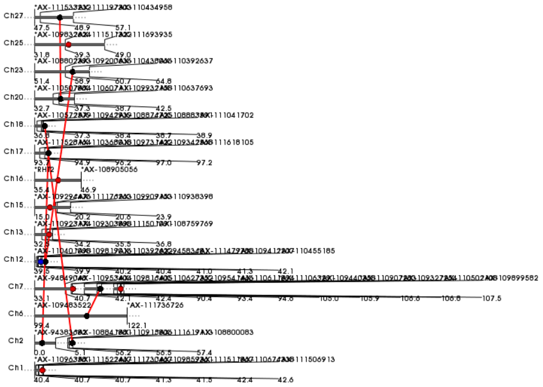

GPW

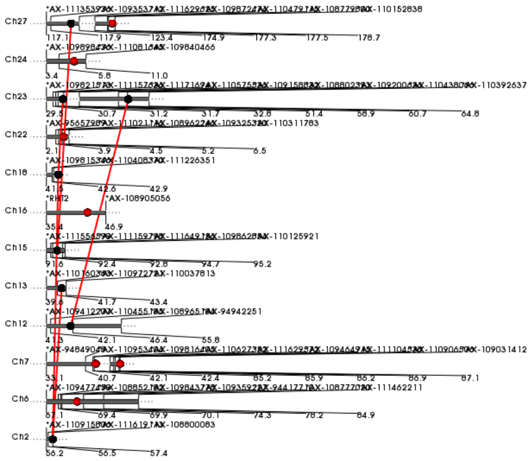

GSW

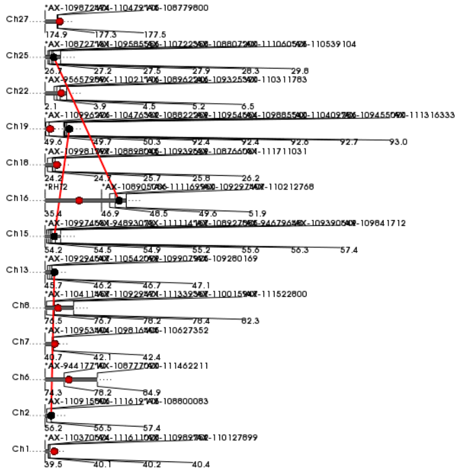

GWW

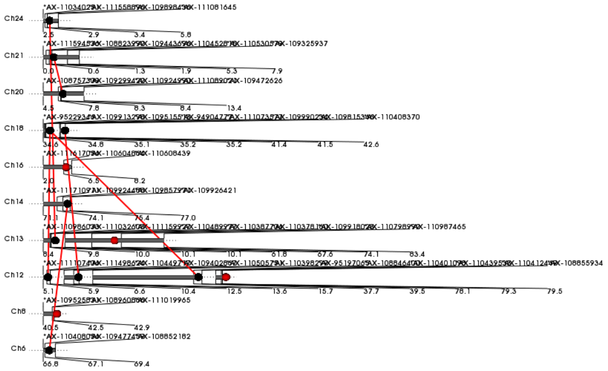

PC

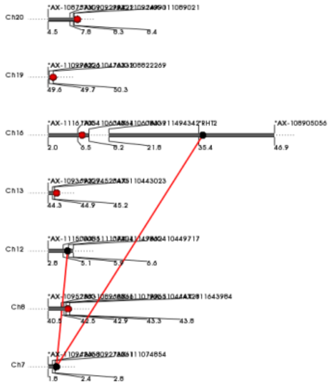

SC

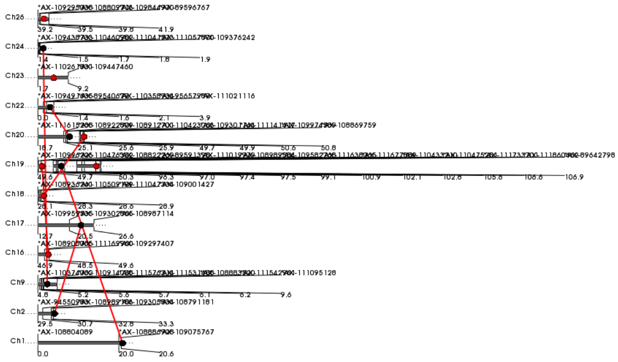

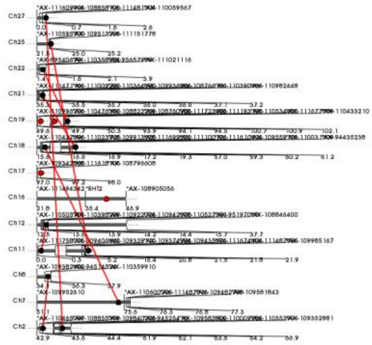

MC

GH

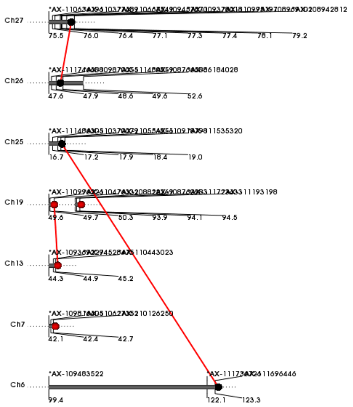

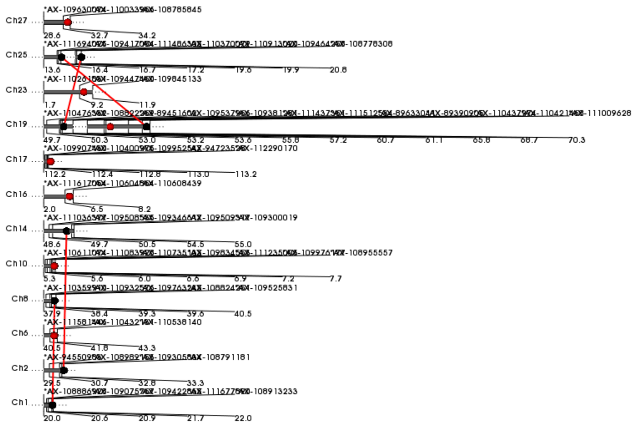

ABS

TW

Figure S4 Diagram of the QTL identification for ten measured traits by QTLNetwork 2.1 in the combined environments. The horizontal lines represent chromosomes; the circles on the chromosomes represent the loci with additive or epistatic effects; the red lines between two circles represent the epistatic interaction between two loci; the red circles represent the loci with additive effect; the black circles represent no additive-related effects.

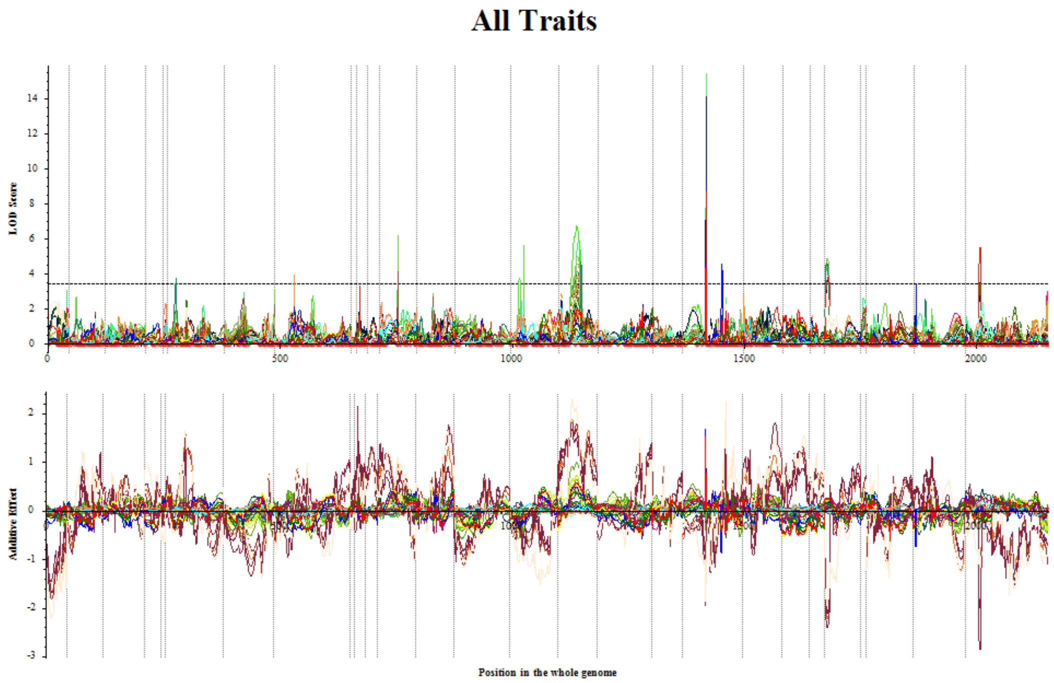

Figure S5 Diagram of the additive QTL identification for ten measured traits by IciMapping 4.1 in the individual environment. The horizontal lines represent the LOD threshold 3.42.

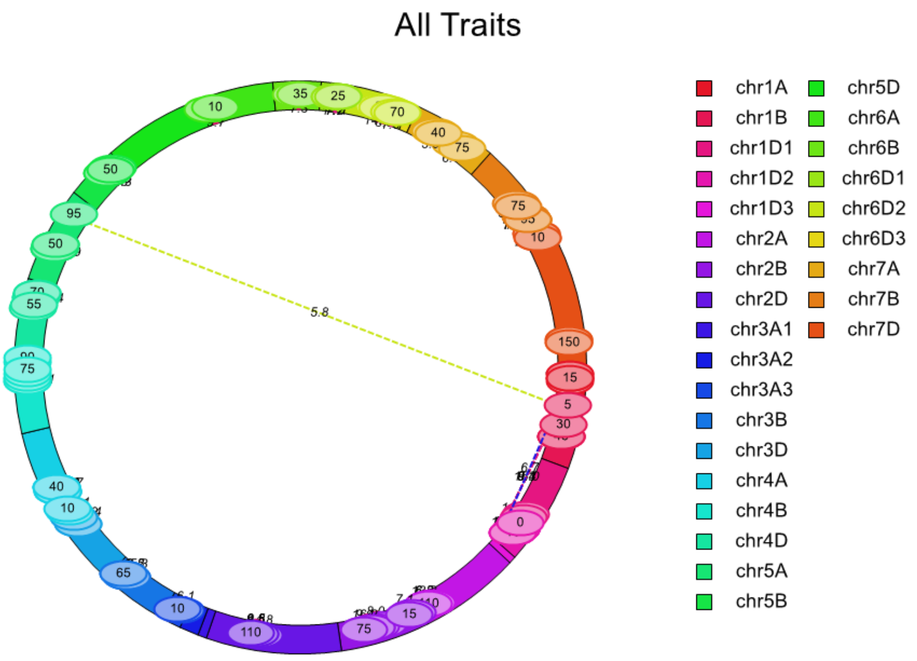

Figure S6 Diagram of the epistatic QTL identification for ten measured traits by IciMapping 4.1 in the individual environment.
